# Analysis of Continuous Prevalence Survey of Healthcare-Associated Infections Based on the Real-Time Monitoring System in 2018 in Shandong in China

**DOI:** 10.1155/2021/6693889

**Published:** 2021-06-11

**Authors:** Jian Sun, Wen Qin, Lei Jia, Zhen Sun, Hua Xu, Yiyi Hui, Anman Gu, Weiguang Li

**Affiliations:** ^1^Department of Infection Control, Shandong Provincial Hospital Affiliated to Shandong First Medical University, Jinan, Shandong 250021, China; ^2^Department of Infection Control, The Affiliated Hospital of Qingdao University, Qingdao 266062, China

## Abstract

**Background:**

Healthcare-associated infection (HAI) is a serious threat to the safety of patients worldwide. The prevalence survey is widely used to explore and study the characteristics of HAI. However, the annual continuous prevalence survey of hospital-acquired infections has not been reported so far.

**Aim:**

This study is aimed at examining the occurrence and development trend of HAIs dynamically and accurately.

**Methods:**

An annual continuous HAI prevalence survey based on the real-time monitoring system was conducted in representative hospitals from different regions in Shandong in China. *Findings*. A total of 64 hospitals participated in the survey, and 2,741,433 patients were monitored in 2018. The highest prevalence of HAIs in Shandong was 3.83% (February 15), the lowest was 1.85% (February 28), and the average was 2.45%. The percentile distribution of prevalence of HAIs in this study was as follows: P10, 2.23%; P25, 2.31%; P50, 2.41%; P75, 2.55%; and P90, 2.73%.

**Conclusion:**

This study dynamically and accurately showed the occurrence and development trend of HAIs in Shandong in 2018. The results of this study can be used as a reference for the HAI prevalence survey in various medical institutions in Shandong and provide the basis for the regional HAI prevention and control strategy.

## 1. Introduction

In recent years, the outbreak of healthcare-associated infections (HAIs) has been frequent. According to relevant reports [1, 2], a variety of risk factors were related to HAIs, including advanced age (>65 years), ICU stay, indwelling central catheter, undergoing previous surgery, emergency admission, hospital stay longer than seven days, indwelling urinary catheter, and endotracheal tube.

HAIs increase the economic burden and even lead to the death of patients [3]. Nearly 2 million people suffer from HAIs in the United States every year; the direct economic loss caused because of HAIs is at least $28.4 billion per year, and the indirect economic loss is at least $12.4 billion, including early death and productivity loss [4]. About 5000 people die each year due to HAIs in the United Kingdom [5]. HAIs have increased the total cost of medical treatment by 70% in China [6].

The HAI prevalence survey refers to the survey of the actual situation of HAIs in a specific population in a specific period. It is a useful surveillance method to examine the occurrence of HAIs; find out the change trend; understand the HAI rate, the sites of HAIs, and the distribution of HAI pathogens; and timely monitor the high-risk groups and high-risk factors [7]. Moreover, it can detect the defects in daily monitoring of HAIs and provide a basis for formulating reasonable prevention and control measures.

However, the traditional bedside investigation and the medical record consultation consume a lot of human and material resources. Therefore, carrying out HAI prevalence survey continuously every day is difficult. The informatization of HAI management is an important solution, which can reasonably use hospital resources to do a good job in HAI control, improve the efficiency of HAI management [8], and realize the real-time monitoring of the condition of inpatients, invasive operation, drug use, microbiological detection, and other information. It can improve the accuracy and authenticity of the HAI prevalence survey by automatically obtaining the HAI relevant information.

This study is aimed at examining the occurrence and development trend of HAIs dynamically and consecutively by the HAI real-time monitoring system and provide references for the HAI prevalence survey in various medical institutions in Shandong and the basis for the regional HAI prevention and control strategy. The annual continuous prevalence survey method through the HAI real-time monitoring system has not been used in previous studies.

## 2. Methods

### 2.1. Survey Design and Participating Hospitals

An annual continuous HAI prevalence survey was designed based on the real-time monitoring system. A total of 72 representative hospitals from different regions in Shandong were invited to participate in the survey. Finally, 64 hospitals (88.89%) participated in the survey. All hospitals participated voluntarily, and an agreement was signed with each hospital. These hospitals were classified as provincial, municipal, and county-level hospitals.

According to the results of the national prevalence survey in 2014 [9], the prevalence rate in China is about 3%. A sample size of 112792 produces a two-sided 95% confidence interval with a width equal to 0.002 when the sample proportion is 0.03 (PASS 11.0). In order to reduce the sampling error and increase the accuracy of the survey and the representativeness of the sample, we actually included 2,741,433.

### 2.2. Early Warning of HAI Cases

The HAI real-time monitoring system collects the relevant data from the hospital information management system, laboratory information management system, radiation information management system, and electronic medical record management system through data access middleware technology. It establishes the dynamic basic database of infection information and realizes the online monitoring of the whole process of patients from admission to discharge. Through the professional screening strategy formulated by the full-time staff, the system automatically carries out multiparameter comprehensive analysis and intelligent identification on the high-risk elements of HAIs and prompts the cases that meet the early-warning standard with red identification. Based on the decision of the full-time staff, the early-warning cases are excluded or become suspected infection cases. The high-risk elements of HAIs are divided into seven categories: general risks, risks related to self, risks related to diagnostic information, risks related to treatment, risks related to laboratory tests, risks related to physical signs, and risks related to interpretation of HAI. General risks include medical record number, ID number, admission time, and discharge time. Risks related to self include date of birth and birth weight. Risks related to diagnostic information include outpatient diagnosis, admission diagnosis, and discharge diagnosis. Risks related to treatment include name of operation, operation start time, and end time of operation. Risks related to laboratory tests include sample name, sample collection time, and etiological results. Risks related to physical signs include temperature, temperature measurement time, and diarrhea frequency. Risks related to interpretation of HAI results include site of HAI, date of HAI, and outcome of HAI. In total, 70 data elements exist as shown in Table 1.

**Figure 1:**
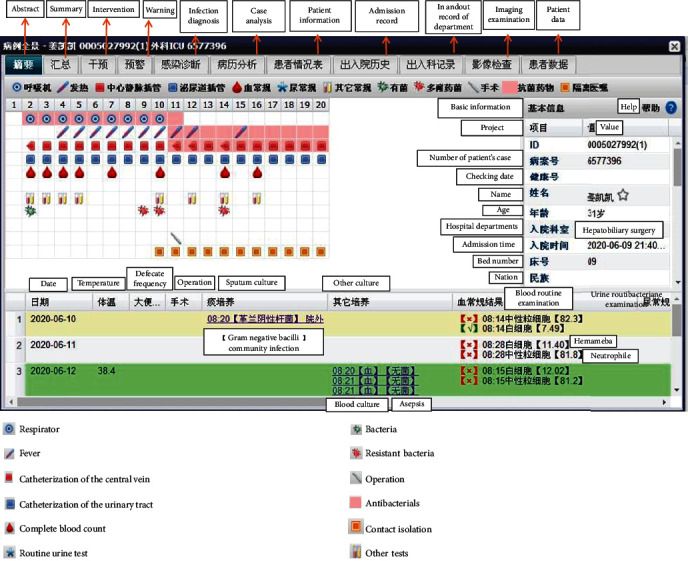
Early-warning interface of the real-time monitoring system.

The early-warning interface of the real-time monitoring system is shown in Figure 1.

### 2.3. Diagnosis of HAI Cases

**Figure 2:**
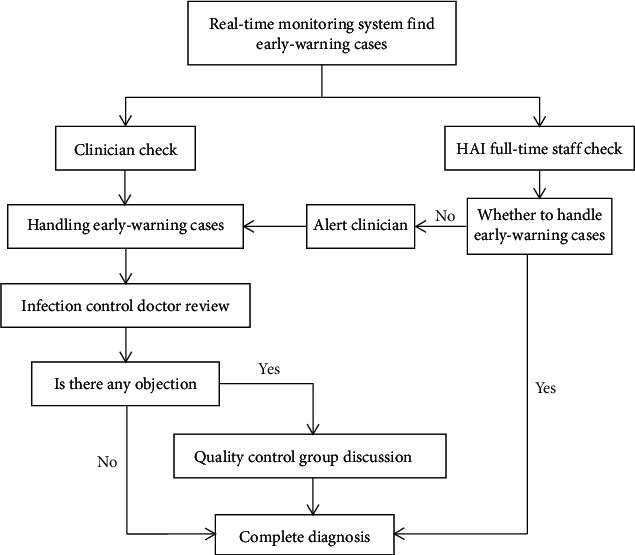
Diagnostic process of infection cases.

**Figure 3:**
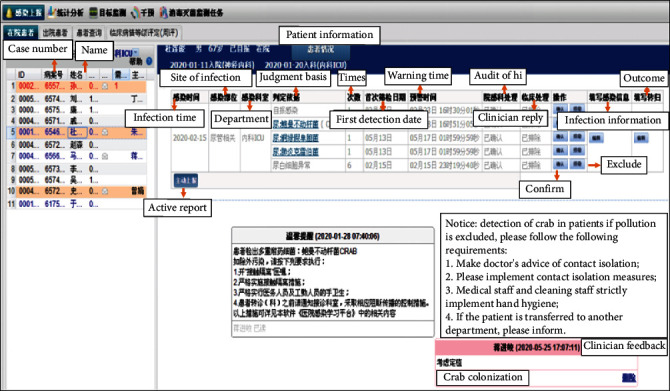
Interface of interactive platform.

The full-time staff of the hospital infection management communicates with the clinicians through the interactive platform in the real-time monitoring system. The interactive platform passes on the identified suspected infection cases to the clinician workstation in real time and uses the red mark for the patient's name and ID number to alert the physicians. The clinicians can enter the platform interface after clicking and confirm or exclude the cases of hospital infection according to the definition criteria established by the original Ministry of Health of the People's Republic of China, which was adapted from the US Center for Disease Control and Prevention (CDC). Once the cases are difficult to diagnose, the clinician and the full-time staff can discuss, communicate, and solve these problems using the interactive platform. The full-time staff can use the platform to timely pass on the diagnosis suggestions, infection prevention and control measures, standard operating procedures, and other contents to clinicians for intervention. The diagnostic process of HAI cases is shown in Figure 2, and the interface of the interactive platform is shown in Figure 3.

### 2.4. Interconnection of HAI Data in Shandong

Most of the existing real-time monitoring systems in China are developed and designed by different manufacturers based on the needs. The compatibility among different systems is poor. The definition, expression, and recording of the same monitoring data have obvious differences. The data between different systems cannot be shared and compared. The Shandong Provincial Center of Hospital Infection Control (SPCHIC) took the lead in developing a basic data set technology in China to solve this problem. The basic data set of HAI monitoring was mainly designed for inpatients, focusing on the identification of main HAI risks of inpatients.

### 2.5. Statistical Analysis

SPCHIC collected the standardized data reported by participating hospitals in 2018 and used the SPSS software, version 22.0 (IBM, NY, USA), for statistical analysis, so as to calculate the prevalence of HAIs every day in 2018 in Shandong. The prevalence of HAIs was the percentage of patients with at least one HAI and was calculated by dividing the number of HAIs by the number of inpatients and multiplying by 100.

## 3. Results

### 3.1. Description of a Monitoring Network of SPCHIC

**Figure 4:**
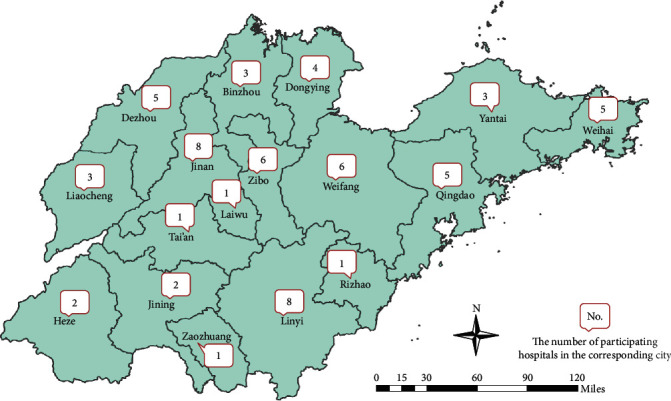
Regional distribution of participating hospitals.

Shandong is a coastal province located in East China. By the end of 2018, it had jurisdiction over 17 prefecture-level cities and 137 county-level administrative regions, with a permanent population of 100.5 million. The gross domestic product of the province is 7647.0 billion yuan renminbi, ranking the third in China. The monitoring network of SPCHIC covers 72 hospitals. Of these, 64 hospitals (including 6 provincial hospitals, 45 municipal hospitals, and 13 county-level hospitals) participated in the survey. The regional distribution of participating hospitals is shown in Figure 4. The total number of inpatients monitored in 2018 was 2,741,433.

### 3.2. Percentile Distribution of Prevalence of HAIs in Shandong in 2018

The percentile distribution of prevalence of HAIs in this study was as follows: P10, 2.23%; P25, 2.31%; P50, 2.41%; P75, 2.55%; and P90, 2.73%. The percentile distribution of prevalence of HAIs in provincial, municipal, and county-level hospitals is shown in Table 2.

### 3.3. Prevalence of HAIs in Shandong from January 1 to December 31, 2018

**Figure 5:**
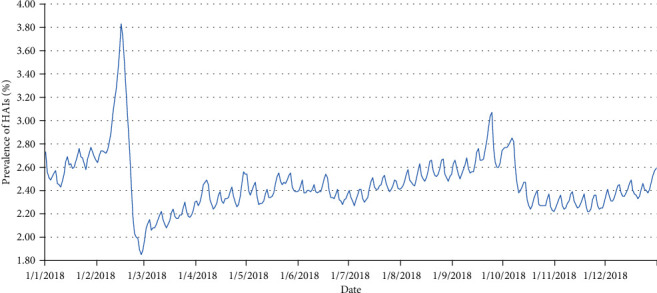
Prevalence of HAIs in Shandong from January 1 to December 31, 2018. This figure directly showed the occurrence and development trend of HAIs everyday dynamically and accurately.

In 2018, the highest prevalence of HAIs in Shandong was 3.83% (February 15), the lowest was 1.85% (February 28), and the average was 2.45%. The details are shown in Figure 5.

## 4. Discussion

The advantage of a prevalence survey, one of the important methods of HAI monitoring, is that it can be completed in a short time and helps understand the characteristics of HAIs comprehensively and quickly [10, 11]. The survey methods are mainly bedside investigation and medical record consultation [12, 13]. The investigators first use a standardized questionnaire to collect the relevant information and then input and analyze the collected data. Traditional bedside investigation and medical record consultation need manual work and input, which usually lead to repeated work and error. These methods are prone to incomplete data extraction, have low efficiency, and cannot achieve real-time monitoring.

In this study, the HAI monitoring system was used to improve the efficiency of HAI management and realize real-time monitoring of infection information of patients with HAI. The real-time monitoring system comprehensively analyzed the infection-related data from the date the patient was admitted to the hospital through the professional screening strategy formulated by the full-time staff. Then, the suspected HAI cases were passed on to the clinician workstation in real time through the interactive platform, and clinicians confirmed or excluded them, so as to realize accurate diagnosis, intervention, and feedback on infection cases. After the diagnosis of HAI cases was completed, the statistical function of the monitoring system was used to calculate the prevalence of HAIs, so as to improve the accuracy, timeliness, and authenticity of the prevalence survey and save a lot of human resources.

Researchers have widely used the prevalence survey, a time-effective epidemiological survey method, to explore the characteristics of HAI. Most of these studies were based on a certain time point [14, 15] or a comparative study of several time points before and after. However, the annual continuous prevalence survey of HAI was not reported. For example, a German study compared the prevalence of HAIs between 2016 (4.6%) and 2011 (5.1%) [16]. In recent years, researchers have carried out repeated surveys for a period of time or consecutive years to explore the characteristics of the changes. For example, a 4-year prevalence study in Italy showed that the prevalence of HAIs fluctuated between 5.5% and 7.1% [17]. The prevalence of HAIs in 135 hospitals in Sweden from 2008 to 2014 showed that the prevalence of HAIs was 7.8%–10.0% [18]. In this study, the real-time monitoring system was used to carry out the annual continuous survey of the HAI prevalence. It could examine the occurrence, development trends, and characteristics of HAI and also serve as a reference for HAI prevalence survey in various medical institutions in Shandong. It could also provide a basis for formulating regional strategies for the prevention and control of HAI.

The average HAI prevalence in Shandong in 2018 was 2.45%, which was higher than that in the Inner Mongolia Autonomous Region (1.65%) and Heilongjiang province of China (1.71%) [19, 20] and slightly lower than that in China (2.67%) [9]. It was lower than the values obtained in some foreign studies on HAI prevalence. For example, in 2018, a multicenter HAI prevalence survey in Italy reported it as 1.7%–30.6%, with an average of 10.3% [21]. A 2016 multicenter HAI prevalence survey from Ghana showed that the prevalence varied from 3.5% to 14.4%, and the total prevalence was 8.2% [22]. In 2016, the first multicenter prevalence survey in four university-affiliated hospitals in Japan reported it as 7.7% [23]. A prevalence survey conducted in Brazil, Colombia, Mexico, and Venezuela, the four Latin American countries, indicated the prevalence as 11.5%, and the survey involving 20 hospitals in Sweden showed it as 13.1% [24].

The percentile distribution of the prevalence of HAIs in this study (P10, 2.23%; P25, 2.31%; P50, 2.41%; P75, 2.55%; P90, 2.73%) was also different from that of China in 2014 (P10, 1.44%; P25, 2.14%; P50, 3.17%; P75, 4.00%; P90, 4.99%) [9], which might be related to many factors, such as the survey method, current patient rate, statistical method, and number and scale of hospitals included in the study.

This study found that in the whole year of 2018, the prevalence during the Spring Festival was the highest (3.83%). The reason is that the Spring Festival is the most solemn festival in China, and the Chinese custom is to have family reunions in major festivals. When the condition allowed, most of the patients chose to leave the hospital and spend the festival with their families. Therefore, most of the patients who were still in hospital during the Spring Festival were those who were hospitalized for a long time and were in critical condition. The HAI prevalence in these patients was higher than the previously reported values. A similar trend was seen during the Mid-Autumn Festival (3.07%) and National Day (2.85%). This study found that in a week's time, the prevalence during the weekends was usually the highest. In China, patients have a right to choose their discharge time once they are cured, and they choose to leave the hospital on weekends. This is because they need the help of relatives and friends to go through the various procedures when they are discharged from the hospital. Obviously, these patients who can be discharged smoothly are the group with low HAI prevalence. However, the HAI prevalence of those who are still in hospital increases correspondingly.

At present, real-time monitoring systems have been widely used in Shandong. Many brands, including Xinglin system, Wisdom system, Blue Dragonfly system, Zongyang system, and Zexin system, are available. In 2014, SPCHIC took the lead in realizing the interconnection of HAI data of major monitoring systems in China through the basic data set technology. The establishment of the basic data set of HAI monitoring is aimed at solving the problem of standardization of HAI data, which includes the definition, category, purpose, format, data source, extraction description, extraction scope, and exclusion scope of various data.

## 5. Conclusions

This study examined the occurrence and development trend of HAIs dynamically and accurately. The HAI real-time monitoring system was used to carry out the annual continuous prevalence survey, which not only improved the efficiency of the survey but also saved lots of human resources. The results of this study can be used as a reference for the HAI prevalence survey in various medical institutions in Shandong and may provide the basis for the regional HAI prevention and control strategy.

## Figures and Tables

**Table 1:** Classification of high-risk elements of HAIs.

	Classification	Number
1	General risks	12
2	Risks related to self	2
3	Risks related to diagnostic information	10
4	Risks related to treatment	20
5	Risks related to laboratory tests	14
6	Risks related to physical signs related	4
7	Risks related to interpretation of HAI results	8
	Total	70

**Table 2:** Percentile distribution of prevalence of HAIs in Shandong in 2018.

Level of hospital	Percentile distribution (%)				
	P10	P25	P50	P75	P90
Provincial hospital	2.35	2.43	2.51	2.64	2.85
Municipal hospital	2.23	2.31	2.41	2.55	2.73
County-level hospital	2.12	2.23	2.35	2.46	2.65
Total	2.23	2.31	2.41	2.55	2.73
China^a^	1.44	2.14	3.17	4.00	4.99

^a^Results of prevalence survey of HAI of China in 2014 reported by Ren et al. [9].

## Data Availability

The underlying data supporting the results of our study can be found in the manuscript.
